# Book Reviews

**Published:** 1819-09

**Authors:** 


### CRITICAL ANALYSIS

OF

### RECENT PUBLICATIONS, IN THE DIFFERENT BRANCHES OF MEDICINE AND SURGERY;

### SELECT MEMOIRS, AND HISTORIES OF CASES;

### In the Literature of Foreign Nations.

---

Πατρίδες ἄρα
Ανδράσιν, ὧ πατραῖς ἄνδρες, ἀγαλλόμεθα.

---

*An Essay on the Means of lessening Pain, and facilitating certain Cases of difficult Parturition.* By W. P. Dewees, m.d. Lecturer on Midwifery in Philadelphia, &c. 8vo. pp. 156. Dobson and Son, Philadelphia, 1819; with the epigraph,

——ut si

Cœcus iter monstrare velit.—Hor.

THE title of this work is the most interesting one that can be given to a medical essay. What can more certainly and forcibly engage the attention of every physician, than a dissertation on the means of lessening the pain and danger of child-birth? The fatality of this state, although not so extensive at present as it was a century or two since, when it was chiefly superintended by female practitioners, is still dreadful. Every attempt, therefore, to lessen its danger, either by a relation of the results of fortunate experience, or by speculative reasoning, must be respectively received with the warmest gratification, and examined with the most assiduous care; though it must be acknowledged that the practice of medicine has not often been directly benefitted by prolusions of the latter kind. The essay of Dr. Dewees comprises matter possessing both of the above characters.

Before we enter into the consideration of the author's view of the proximate causes of the pain and danger of child-birth peculiar to women of civilized life, let us, since speculations on it are now to be indulged, adduce a sketch of that which we have ourselves taken of this subject; not from slight and casual consideration, but from repeated and serious attention.

On contemplating the natural history of man, through his various degrees of intellectual cultivation, from the *Papous* to the most civilized nations of the temperate climates of Europe, we see a progressive increase in the pain and suffering of the female in the act of parturition. Amongst the former, the mother, after the expulsion of her infant without a sigh or a groan, rises to go and bathe it in a neighbouring stream, and immediately assumes her ordinary and active habits of life: in the latter, parturition is an act of long and dangerous suffering, and is fatal to the life of at least one in fifty of those who undergo its pains.

This difference is not chiefly dependant on diversity of anatomical structure; for we find that civilized tribes of a certain race of people suffer the dangers to which we have just alluded; whilst other tribes of the same race, remaining in the state of barbarism, encounter the act of parturition without either grief or fear, although no remarkable difference of formation is evident. This is witnessed in the Tartars and in the Chinese. In the ruder tribes of the former, the female joins the progress of her horde immediately after delivery; whilst the sufferings of the latter on the same occasion are hardly inferior to those of the most delicate European. The Chinese husband may now take to his bed on the birth of his child, but he does not find his wife able to attend on him with the pampering luxuries he then, from custom, may require. It is common at present, in some of the more retired parts of Ireland, for the women who dwell in huts formed only of stone-walls, a thatched-roof, and a door; in which the only furniture is an iron pot, a few knives, and some wooden bowls; containing neither chair, table, bed, nor altar of the Penates;—and who, passing their lives with so little about them to excite variety of ideas, are in such a state of non-intellectuality, that they seem to be guided by what in brutes is termed pure instinct;—it is common for those females to appear without their cabins within a few hours after their delivery, which they have quietly undergone, without fear and without trouble. But, is it thus with those of their countrywomen who inspired their own Anacreon with those ideas of sentimental refinement that are the acmé of human feeling?

It is not, then, to anatomical formation, but to physiology, that we must look for the explanation of this phenomenon.

An ancient philosopher, whose profound wisdom is often rendered obscure by the metaphorical manner in which he expressed his sentiments, seems to have attributed the sorrow of child-birth to the changes effected in women by unrestrained indulgence of the passions, and intellectual cultivation. To this explanation we can only add a few illustrative observations.

It is ascertained that the ganglionic system of nerves supplies the parts subservient to organic life, in which may be included those appropriate to the re-production of the species, with the sensibility necessary for the execution of their functions: excitement of this sensibility is not directly perceived by the sensorium, or, as the French physiologists expressively term it, the *moi*, so as to give rise directly to either pain or pleasure. Thus, notwithstanding the delicate sensibility of the stomach, the digestion of the food is not attended with conscious perception. When this does take place, from the functions of organic or nutritive life, it is because, from inordinate impressions, the sensorium is also excited. Sometimes impressions on the brain are also made by the ganglionic system of nerves, that induce volition without either pain or pleasure, (or it is so slight, perhaps from habit, as not to be very forcible,) as in the expulsion of the urine and fæces.

Excitement of the cerebral system and an effort of volition, appear to be necessary, in persons subservient to our habits of civilized life, for the expulsion of the contents of several of the reservoirs of the excretory matters of the body: it is evident with respect to the bladder

2

and rectum; for, in some cases of apoplexy and palsy, when all the functions of organic life, especially digestion, chylification, and the secretion of the urine, are carried on with the ordinary vigour, retention of the urine and fæces will take place, not from any inordinate material obstruction, but because the sensorium is not excited to direct the act of volition to aid in their expulsion. That the same excitement of the sensorium is necessary to procure the expulsion of the fœtus from the uterus, seems to be evident; but this is effected in the savage, as well as in wild brute animals, with but little or no pain, any more than the evacuation of the fæces.

It may be doubted whether or not this excitement of the cerebral system in the cases above mentioned, occurs in the savage; that is, whether or not it be a natural law in the animal economy; since the urine and fæces are sometimes evacuated during sleep, without dreaming, by children; and uterine action is known to go on in parturient women during sleep, (it should be here observed, that this is not accompanied with any action of the abdominal muscles,) and fœtuses have been expelled from the uterus after the cessation of the functions of animal or relative life. This question does not affect our explanation, since such a connexion of cerebral with ganglionic nervous energy does take place in ordinary circumstances in civilized life; and if it be not a primitive natural law, the influence of the cause of the pains of child-birth we are about to designate, is only the more forcibly shown. This is the inordinate sympathy and synenergy that civilization has produced between the nervous systems of organic and of relative animal life. Impressions on the former of these, that produce but little excitement of the latter in the savage, or one whose intellectual faculties have not been much developed, cause intense suffering and action in persons whose cerebral system has acquired that superiority in the human economy, which is produced by our habits of society and our mental cultivation. This is evident in a multitude of facts. It may here be sufficient to point out the extreme bodily injury savages will undergo, without showing signs of much pain, and their almost absolute freedom from insanity; whilst a little disorder of the stomach or liver, in persons in a state of civilization, especially those in whom this is carried to the greatest degree of refinement, will frequently be productive of mania.

Applying this principle to parturition, we would say, that the call made by the uterus in that act on the cerebral system, which in the savage is either not experienced, or so slightly perceived as only to cause an effort with no more sensation of pain than what ordinarily takes place in the expulsion of the fæces, produces in the woman of civilized life, in whom indulgence of the passions and intellectual cultivation have caused an inordinate development of cerebral energy, the pain and other serious accidents they so generally endure.

This, then, is the view we are disposed to take of this phenomenon: could we indulge in a more detailed explanation, it might be rendered more apparently correct; but we are contented with pointing out to others what we believe to be an interesting course of enquiry, and one that, if pursued, might perhaps lead to some useful results. Before we conclude, it may be useful to observe, that these notions are powerfully supported by the observations of Mr. Power; whilst they seem, in return, to elucidate the view he has taken of the same subject.

We will now consider the essay of Dr. Dewees; and, in the first place, the explanation he advances of the same phenomenon. He commences with remarking, that a change has obviously taken place in civilized life, by which parturition is rendered an act of difficulty and danger; and he states his object in this essay to be, "to show in what some of these changes consist, and how to ameliorate them."

The causes of difficult and painful labours, he continues to remark, may be divided into two general heads: those arising from some imperfection of the pelvis, and those which interrupt the natural and healthy functions of the soft parts. It is to the latter which his attention in this essay is confined.

The author, in the first instance, gives a brief account of the opinions of Dr. Hunter, Malpighi, and Ruysch, respecting the arrangement of the muscular fibres of the uterus, notices their discrepancy, and then observes, that the correctness of neither of those descriptions has been satisfactorily proved. Since then, he continues, "anatomists have never been able with their knife to clear up this point, we must have recourse to other means to satisfy ourselves: the only certain one, it appears to me, is, to deduce them from the actions of the uterus itself; this at least warrants two distinct sets, the one circular, the other longitudinal."

This, it must be well known, was the opinion of Vesalius, though he also supposed the existence of transverse fibres.

The basis of the arguments of Dr. Dewees is by no means a valid one for such a deduction, and will not be so admitted at the present time, when physiologists are not disposed to limit the property of contractibility to muscular fibres; and it is not well ascertained that the chief actions of the uterus are expressly longitudinal, and transverse or circular. Many observations, especially those made when the placenta has been attached to unusual parts of the uterus, and when there has been two or three children in it at the same time, seem to show that, like the stomach, it performs numerous varieties of action, according as it is irritated in one part or in another. Such actions are shown to take place in the stomach by the expulsion of substances from it, by vomiting, that are not adapted for digestion, whilst alimentary matter is retained.*

Let us for a time, however, admit of the supposition of Dr. Dewees, since the establishment of this forms the basis of his dissertation; and, if we do not acknowledge it, we cannot readily proceed with him in his enquiry.

Taking for granted, then, the existence of longitudinal and transverse muscular fibres in the uterus, the author next proceeds to state, that "he cannot help regarding the neck of the uterus as a distinct and independant part from the body and fundus, and as having its

* See our review of Dr. Lallemand's Thesis in the fortieth volume of this Journal. Many curious facts of this kind are there noticed.

own peculiar laws and actions; and that this separation of powers is absolutely necessary to the explanation of some of the pheuomena exhibited by health and disease, and the influence of certain agents on these parts.

This susceptibility to diseased action cannot be well disputed, and is indeed generally admitted. Dr. Dewees next adduces a statement of all the circumstances, which he considers most concur to produce parturition with the greatest possible regularity. These are,

"First, there must be a subsiding of the abdominal tumor; and, if at this time a finger be introduced through the os tincæ, we shall find the membranes alternately tense and relaxed: these circumstances are owing to the uterus now beginning to contract, and forcing the presenting part into the pelvis.

"Secondly, there must be a secretion of mucus from the vagina: in some instances this flows from it, several days previous to the onset of pains; but, for the most part, it only happens a few hours before they are felt. This mucus is secreted from the surface of the vagina, and perhaps from a portion of the neck of the uterus. Why these glands are excited to this duty may perhaps be difficult to explain, but their action appears intimately connected with a certain state or condition of the os tincæ: thus we find, *cæteris paribus*, this secretion to be most abundant where there is the greatest disposition in the mouth of the uterus to dilate or relax; and with this is connected the same disposition of the perinæum. It must be observed, that the secretion here spoken of must not be confounded with leucorrhœa, as this discharge is by no means favourable to this effect.

"Thirdly, the mouth of the uterus must yield easily, that the contractions of the body and fundus may not be exerted for too long a time unavailingly. The dilatation of the mouth of the uterus, when best performed, is either before or very quickly after the painful contractions of the uterus have taken place: this, in general, is done without the mechanical aid of the contents of the uterus.

"Fourthly, the body and fundus must contract with sufficient force, to make the child pass through the pelvis.

"Fifthly, the perineum must unfold without much or any mechanical force, that the child may not be detained in passing through the os externum.

"Sixthly, there must obtain between the fœtus and pelvis a proper proportion, and the former must be well situated, that it may derive every advantage from the circumstances just enumerated."

In his observations on the dilatation of the orifice of the uterus, we meet with nothing either novel or particularly remarkable: his chief object is to show that, "in the most natural and favourable cases of labour, the mechanical power of the ovum has no influence in dilating the mouth of the uterus." This is generally admitted, and Dr. Dewees might have spared his disquisition on the description of Baudelocque; for that author, in his own language, advances the same doctrine. He next considers "*the different kinds of contraction of the uterus.*"

"First, the longitudinal contraction: this is performed by the fibres of the uterus, so called, or those fibres which run from the fundus to the neck; it serves to shorten the uterus in the direction of these fibres; consequently, to expose its contents more and more, by making them approximate the mouth; and this will be in proportion to the diminution of resistance at this part, and the force with which these fibres may act.

"The circular contraction. This action is performed by the fibres so named; they, as it were, run round the uterus, commencing at the fundus, and terminating in the circle forming the neck; they tend to diminish the capacity of the uterus in the direction of its transverse diameter; consequently, have little or no immediate agency in expelling its contents.

"The simple contraction, or when either of these sets of fibres act separately, as, before labour especially, when the finger is introduced through the os tincæ, we find the membranes alternately tense and relaxed. In this case, we presume, the longitudinal fibres act alone, as there is no stiffening of the circle forming the mouth; or, as when the waters have been evacuated, the uterus is made to embrace its contents, and no pain for a long time is produced: we suppose, in this instance, the circular fibres act alone, as there is no effort to expel the contents of the uterus, which would not be the case did the longitudinal fibres co-operate with them.

"Compound contraction, or when both sets of fibres act. Their united action is proved, we conceive, when there is a hardening of the mouth of the uterus, and an evident depression of this viscus, with its contents, into the lower part of the pelvis.

"Tonic contraction. By this we understand that uniform action which the uterus exerts to reduce itself to its original size: this appears to be the effect of all the fibres folding themselves up after the distracting cause is removed.

"The spasmodic, or that contraction of the uterus which is for the most part accompanied with pain. It must be remembered, however, that pain does not necessarily belong to this species of contraction, since some women are delivered without it. We should therefore, agreeably to this fact, rather call this species the alternate contraction of the uterus, as it has a greater or less interval between each contraction. When this action is best performed, it is, we presume, chiefly by the longitudinal fibres.

"It may here become a question," continues the author, "how are the fibres of the uterus enabled to perform this alternate contraction? since we know that a muscle, after having contracted, cannot repeat that contraction, without being first relaxed, and then elongated." We shall not follow him in his remarks on this point; it has no direct connexion with the question under consideration, and we are desirous to give as clear and direct an exposition of his arguments in favour of his hypothesis, as his desultory manner will permit. It should be remembered, that the existence of muscular fibres in the uterus is not determined; that membranous contraction will proceed without intervals of relaxation; and that the *tonic* and *spasmodic* mode of action, which the author enumerates, seem, with the aid of the abdominal muscles, sufficient to explain all the consequences observed in the mechanism of the act of parturition.

The author then considers "*the relative strength of the different sets of fibres*;" he says,

"We have already observed, that the longitudinal fibres were stronger than the circular: our reasons for thinking so are,

"First, that, were they of equal strength in all parts of the uterus, delivery could not take place, since the circular fibres would embrace the body of the child, and thus retain it; their action being, as we have already said, to diminish the uterus only in its transverse diameter, consequently, is at right angles with the longitudinal.

"Secondly, that, when from any circumstance the power of the circular fibres is increased, either absolutely or relatively, there the labour does not advance; therefore, the circular fibres are not to be considered as directly instrumental in expelling the child.

"Thirdly, as the circular fibres, from the direction of their action, do not immediately contribute to the advancement of the child, they must be considered as the weakest set, since delivery takes place without their direct aid: the longitudinal fibres have then not only to move the child, but overcome their resistance.*

"Now, let us apply these facts to the explanation of the dilatation of the uterus and the progress of labour.

"When the woman has carried her child to the full time of gestation, that process termed labour must ensue, that she may be enabled to part with it; for this purpose one part of the uterus must yield or dilate, while another must contract. The uterus is closed at bottom, and maintained in that situation by the contraction of the muscular fibres of its mouth; but these must relax, that the child may effect its escape. We must therefore regard the circular and longitudinal planes of fibres, as a kind of antagonist muscles to each other. The longitudinal fibres yield more willingly to impulse from within the uterus than the circular during gestation, owing perhaps to their greater length, or perhaps greater laxity:† they continue to yield until they are so much upon the stretch as to induce a disposition to contract; this they eventually do. The circular fibres, on the other hand, from their greater rigidity, most probably are put immediately upon the stretch: they therefore have a constant stimulus to excite their contraction; hence the mouth of the uterus keeping closed. But, so soon as the longitudinal fibres become uneasy, from distention, they become refractory, and will yield no more without resistance; they then contract, and continue to do so until the stimulus of distention becomes still more powerful, which eventually brings on the period of labour. By the contraction of the longitudinal fibres the length of the uterus diminishes: this puts the circular upon the stretch, since the uterus

* This is particularly the case in the instances where the membranes are prematurely ruptured: the uterus, by virtue of its tonic contraction, accommodates itself to the various inequalities presented by the child's body, and some of these are eminently calculated to retard labour; yet the longitudinal fibres overcome them.

† Hence perhaps the lengthened form of the uterus.

cannot diminish in one direction, while the mouth of the uterus remains shut, without augmenting in another; therefore, the circular fibres are a little distracted, and they immediately co-operate with the longitudinal, and force the uterus, with its contents, lower into the pelvis. In this instance, what we have termed the compound action of the fibres, takes place: this is proved by the edges of the mouth of the uterus stiffening during the contraction.

"This kind of action is reciprocated for some time, but the circular fibres eventually yield to the influence of the longitudinal; first, from their having expended a portion of their power in maintaining a state of contraction so long; and, secondly, their being absolutely the weaker fibre. Hence the circular fibres which constitute the neck relax, and hence the dilatation of the mouth of the uterus."

After some adventitious remarks on the secretion of mucus, and the yielding of the external parts, the author proceeds to treat of "*the contraction of the fundus and body of the uterus*." We wholly transcribe his observations on this subject.

"That the uterus may be enabled to expel its contents, as we have already said, the fundus and body must contract, while the mouth must relax. We have endeavoured to show how the latter was effected; let us now, for a moment, attend to what must be done by the fundus and body, that delivery may take place.

"When the mouth of the uterus is sufficiently dilated to allow the child to pass through it, the fundus and body must continue to contract: this contraction is of two kinds, namely, the tonic, and the spasmodic or alternate. The tonic contraction is chiefly performed by the circular fibres: by this contraction the whole of the internal surface of the uterus is applied to the body to be moved, and the longitudinal fibres, by this means, are brought more closely into contact with it, and of course are enabled to act with more effect. This perhaps is the chief use of the circular fibres at this period of labour; as they do not, in any instance, directly contribute to the advancement of the child, as we have already observed, and shall now endeavour to prove more fully.

"We shall relate some circumstances attending the contraction of the uterus, which will deserve notice, and to the truth of which every accoucheur will bear testimony.

"First, that a considerable degree of contraction may take place in the circular fibres without producing pain: thus, after the evacuation of the waters, and the uterus is closely applied to the body of the child, even to a degree that would render turning impracticable, yet no pain is felt.

"Secondly, when this contraction is violent, it throws the uterus into inequalities, and sometimes divides it like an hour-glass: this obtains in a degree before the birth of the child, but more especially after it, and before the expulsion of the placenta. In these instances the contraction is obstinately maintained, but no pain is felt.

"Thirdly, if the finger be applied to the presenting part during the continuance of this contraction, it is not found to advance.

"Fourthly, when this constriction is most violent, the longitudinal fibres act, for the most part, with more force; since they are not only obliged to effect the delivery, but also to overcome this additional resistance: another proof of their superior strength.

"Fifthly, this stricture continues without any intermission, as far as we can determine, for hours; but this does not suspend the painful and alternate contractions of the other fibres, nor does this constriction relax during this alternate contraction; therefore much time is lost, and much pain endured, from this circumstance: it however yields eventually, and from the same cause and in the same manner as the mouth of the uterus does at the commencement of labour.

"Sixthly, when the painful contractions take place, the presenting part is pushed lower into the pelvis; but, when this ceases, it most frequently recedes a little.

"From these facts, the following inferences, we think, are deducible:

"First, that the circular fibres may contract to almost any degree, without being attended with pain.

"Secondly, that their contraction alone, however violent, does not forward the child.

"Thirdly, that they do not possess the power of alternate contraction in the same degree as the longitudinal fibres; and, that they may exert this power, it is necessary at first to have them distracted by some force or other.

"Fourthly, that the pain felt during labour must, in a great measure, if not entirely, depend upon the contraction of the longitudinal fibres.

"Fifthly, that the changes the uterus has suffered from civilization and refinement, must be chiefly confined to its longitudinal fibres.

"From what has been said, then, the spasmodic or alternate contraction of the uterus appears to be nothing more than an increased effort it makes to overcome the obstacles opposed to its progress to a state of vacuity; and that the pain attending these contractions depend upon certain physical changes which the longitudinal fibres have undergone from the causes just mentioned. Why a particular set or given direction of fibres should have suffered more than another, may be impossible to determine; but that they have, we believe to be most certain. This change, however, is by no means confined to the uterus, as every straight muscle of the body appears to have participated with it; since it is admitted that the man of the civilized world has lost much of his original strength. On the other hand, the circular muscles, as far as we can determine, have lost nothing of their primitive power; since it is more than probable that the various sphincters,* among which we may reckon the circular fibres of the mouth of the uterus, perform their duty as effectually and as powerfully as in the time of our first parents.

"Do not these facts emphatically account for women, who have suffered these changes, having more tedious and painful labours than those who have not undergone them? as the women of savage nations, the women of Calabria, &c. and without the necessity of having re-

* The heart, or intestines, may also be included.

course to a physical necessity, derived from the erect position of man, or the peculiar construction of the pelvis?"*

All the postulates in the foregoing arguments are mere suppositions, not rendered at all probable by precise observations. The only facts that seem to be well established are these, that the uterus may be partially excited to contraction, which contraction does not so well tend to expel its contents as that which occurs when it wholly embraces them, as the hand does a ball; and that a certain degree of contraction may take place in it, and that not to be easily overcome, without producing pain: in this case, it seems, the cerebral system is not excited, for no action takes place in the abdominal muscles, which always occurs when that is excited; and it is therefore easy to understand why, during this partial and merely *tonic contractility*, but little or nothing is done towards the expulsion of the fœtus. Whether a particular and direct longitudinal action of the uterus ever takes place, and, if so, if it be always accompanied with pain, remains to be decided; and, we repeat, the author has not rendered his assertion at all probable. What he considers as the expressly longitudinal action of the uterus, is, very likely, merely a continuous grasp of the whole organ, which, through the medium of the cerebral nerves, produces a concurrent action of the abdominal muscles, and is thus so particularly efficacious in forwarding the progress of the fœtus. The reason why the fœtus recedes a little on the cessation of this "*longitudinal action*," is, that the abdominal muscles have ceased to act, as well as perhaps, in some measure, the uterus.

The conclusion which the author draws from all these pure suppositions, is not a little singular, and his corollaries to prove it are not correct. We have no evidence of the existence of that want of balance between the longitudinal and circular muscles of the body; and our women who are engaged in active exertions,—for instance, the stout and hardy Welsh women who visit the markets in the midland counties of England with their agricultural produce,—are much stronger than most female savages; whilst they suffer as severely during labour as any whose minds are equally cultivated by social intercourse. But, we suppose, the patience of our readers must be exhausted ere this, at seeing medicine so sported with, by having visionary data, inaccurate arguments, and almost ludicrous deductions, intruded in the place of that logic, which this science has at length effectually claimed, and which every judicious physician is disposed to revere.

The author, after this, treats the subject of difficult parturition in a more practical manner. But, as we do not comprehend his principles, we shall not enter on the consideration of the application of them. Every thing is reduced to "*rigidity*." He says,

"The cause of pain and difficulty, for the most part, depends upon a certain condition of the soft parts that are subservient to labour; we shall therefore consider this subject under the following heads:

"First, where rigidity of the mouth of the uterus depends on the circular fibres maintaining their contraction for too long a time, but where no inflammation attends.

---

* See Osborne's *Essays*; also *Med. Mus.* vol. i. p. 270.

"Secondly, where the rigidity is attended with inflammation.

"Thirdly, where the rigidity arises from previous local injury, either from mechanical violence, or from inflammation, and its consequences.

"Fourthly, relative rigidity, or where it proceeds from disproportionate powers between the longitudinal and circular fibres.

"Fifthly, tonic rigidity, or where the circular fibres remote from the mouth embrace the body of the child too powerfully."

The remedial measures which the author examines the efficacy of, are opium, the warm bath, and blood-letting. On these subjects he does not appear to us to have advanced any remark of particular novelty, except the inculcating a very free use of blood-letting to alleviate this *rigidity*. The work terminates with some observations on the efficacy of ergot (the *secale cornutum*) in cases of "*difficulty from want of force in the uterus*," &c. We shall not now dwell on this subject, since this remedy in general, and his opinions respecting its use, have been already noticed at length in various parts of this Journal.

---

*A Memoir on Inflammation of the Veins.* By G. BRESCHET, M.D. Prosector to the Faculty of Medicine of Paris.

(*Continued from p.* 166.)

WE have now to adduce the general conclusions of M. Breschet on the pathology of inflammation of the veins, and the appropriate measures for the relief of that disease. As these consist of observations rather than opinions, and are drawn up in a concise and perspicuous manner, we shall give them in detail, and without any particular remarks, as the accuracy of the author's descriptions, and the utility of his labours, must be sufficiently apparent. Dr. Breschet observes, that

"Inflammation of the veins may be either idiopathic, sympathetic, symptomatic, or metastatic.

"*Physiological characters of inflammation of the veins.*—The signs of inflammation of the veins are not always easily recognized; particularly if the inflammation is seated in the small vessels, or even in the trunks, when they are situated in the visceral cavities.

"1°. *Local characters.*—The consideration of the precursory circumstances; pain in the course of the veins; swelling of the adjacent cellular tissue; sometimes redness and tension of the skin; its sensibility and shining appearance in the passage of the vein; and afterwards a knotty, tense, and painful cord, in the particular direction of the vessel; are the most ordinary phenomena of this inflammation. If a wound exists, there may be an evacuation of blood altered from its usual appearance, or of pus more or less thick in consistence, from the orifice of the vein. In the latter case, soon after the lesion of the vessel was effected, there is pain in the wound; swelling comes on; the wound does not unite by the first intention, its edges become hard; or, if it has cicatrized, an abscess forms under the cicatrix, and soon destroys its continuity. The pains which occur in the course of the inflamed vessel are more particularly directed towards the heart than towards the extremities; they are augmented by pressure, and they have sometimes been supposed to be rheumatic. A sensation of burning heat follows exactly the same direction as the pain.

"2°. *General characters.*—It is rare that inflammation of a vein, especially if it has existed some time, is not accompanied with disturbance of the system, or a real febrile state, the intensity of which varies according to that of the inflammation, the extent of the disease, its seat, the importance of the vessel, and the tendency of the local affection to one or other mode of termination. It should be remarked, that many physicians have observed in these circumstances the phenomena of typhus; and I have myself found, in many subjects who had fallen victims to typhus, evident traces of inflammation in the encephalic veins and in the venous sinuses of the cranium. The duration of inflammation of veins is occasionally very short; most frequently, it is of rather long continuance: and it appears, from the observations of M. Fizeau, that this phlegmasia may be remittent, presenting actual paroxysms of exacerbations of the symptoms.

"Inflammation of the veins may be confounded with that of the arteries, the lymphatic vessels, or of the nerves. In the first case, the pain and the other external characters are directed from the point where the artery has been injured towards the branches of the vessel. In the second case, the pain may extend in the direction towards the heart, but the lymphatic glands swell, become painful, and the skin often presents two or three red waving lines, which appear shortly after the lesion or pricking of the vessel; and it is not long before tumefaction of the whole member takes place.

"Lastly, if the nerve be the seat of the malady, the pain is propagated from the situation of its origin to a common centre, and especially from the situation of its ramifications: it is instantaneous; it immediately follows the puncture; and this pain has varieties of character, as we observe in the neuralgiæ, which are often only chronic inflammations of the nerves.

"*Anatomical characters of inflammation of the veins.*—Inflammation of the veins may terminate in several ways. We do not possess well-authenticated facts to prove that it, in any cases, terminates in resolution; and that the vessel, being restored to its former state, executes the functions for which it was primitively adapted. Union of its parietes, obliteration of its cavity, suppuration, ulceration, and gangrene, most commonly take place. Thus, phlebitis appears under the following forms: 1°, adhesive inflammation; 2°, suppurative inflammation; 3°, ulcerative inflammation; 4°, gangrenous inflammation; 5°, eliminatory inflammation.

"1°. *Adhesive inflammation of the veins.*—John Hunter and M. Dupuytren think that adhesive inflammation may exist in veins; and it is indeed by that, that wounds of these vessels are healed in four-and-twenty or six-and-thirty hours. We must, however, distinguish the cicatrization of the external tunic of veins from that of their internal membrane: the former, when it is divided, unites in the same manner as wounds of the cellular tissue; but this is not the case with the adherence of the internal membrane: it takes place more remotely and with more difficulty, from inflammation less readily affecting this tissue. The cicatrix of solutions of continuity of the parietes of veins remains but weak for some time, and is easily torn. Twelve or twenty-four hours after blood-letting, we can, by slight percussion or by distension, destroy the union that has been effected. It is even by these that the adhesive inflammation is changed into the suppurative, and is propagated to a greater or less extent in the cavity of the veins, and sometimes to the heart.

"John Hunter, however, considered the adhesive inflammation of veins as a mean of arresting the progress of the inflammation; and he says, that, by compression excited on the sides of the veins, we may cause them to adhere, and limit the extent of the disease, by preventing the pus formed below the adherence extending further towards the heart. Mr. Travers believes, on the contrary, that this adhesive inflammation is rare in veins, that a considerable degree of irritation is necessary to provoke it, and that, when inflammation is developed, it passes rapidly to suppuration; or else that a large quantity of coagulable lymph is effused into the vessel, which fills up its calibre, obliterates it, and at length becomes adherent to its parietes: but here there is, properly speaking, only adhesive inflammation, only that there should not be so abundant an effusion of coagulable lymph.

"The little disposition to adhesive inflammation offered by the internal membrane of veins, seems to oppose the analogy that has been supposed to exist between that and the serous tissue.

"2°. *Suppurative inflammation.*—It is very common in veins: the greater part of the observations that we have referred to, demonstrate its existence. This species of inflammation has a great disposition to extend further and further on the affected surfaces, and the pus mingled with the blood in its circulation with the continuity of the tissues, is a powerful agent in its propagation. It is in these circumstances that the general phenomena assume their severity of character, and that the state of the patient becomes calculated to excite the most serious alarm. If the inflammation diminish, the pus, from ichorous, serous, or white and consistent, as it had been, is changed for an albuminous fluid, and secondary adherence or obliteration of the vessel is the consequence.

"3°. *Ulcerative inflammation.*—There are many authentic instances recorded of ulcerative inflammation of the veins. M. Portal mentions having found, in the body of a woman, an ulceration of the superior vena cava. Mr. Travers cites a case, where the internal jugular vein was obliterated by the pressure of a tumor situate on the right side of the trachea, and covering the large vessels. The patient, towards the termination of his existence, had voided blood mixed with pus, by the mouth and by the anus. On dissection of the parts after death, the tumor was found to contain gangrenous cellular membrane and blood in a state of putrefaction. The internal jugular vein was filled, to a certain extent, with a coagulum of blood; but below this there was an ulceration that had established a communication with the sac of the tumor, so that the blood which came from the head passed in part into the cyst; there was an ulceration also that had induced a communication of the cyst with the œsophagus, so that the contents of the tumor could pass into the intestinal canal.

"It has been frequently observed, that, if wounds of the veins have not united by the first intention, the suppuration that is formed either in the tunics of the vessel or in the adjacent tissues, was soon accompanied with inflammation of the edges of the wound; that the opening gradually increased in size; and that it was not rare to see the whole circumference of the vessel destroyed by this vessel, and the continuity of its canal cease to exist, by the progress of the ulceration. Lastly, many instances of ulceration of the internal membrane of veins have been observed, but without the perforation of the whole thickness of the parietes of the vessels.

"4°. *Gangrenous inflammation.*—The frequency of suppuration of veins leads to a belief that gangrene might readily affect these vessels: observation, however, demonstrates the contrary. The veins and arteries long resist the effects of the gangrene which destroys all the tissues in the midst of which they may be situated. These vessels are, however, not in a healthy state; inflammation has seized them, and an abundant quantity of albuminous matter, mingled with a little blood, fills them, obliterates the canal for a greater or less extent, and thus opposes the occurrence of hæmorrhage. At length, the vessels, isolated on all sides by the destruction of the other tissues, and no longer receiving nourishment, die by a sort of inanition; a process is set up in some point, but always below the termination of the coagulum; the part of the vessel despoiled of its adjacent parts is separated; and this inflammatory process, by which parts deprived of life are separated from others, is that which I term, after M. Dupuytren, the *eliminatory inflammation.*

"5°. *The eliminatory inflammation.*—When a ligature has been placed on a vein or an artery, it is by this eliminatory inflammation that the vessel is separated throughout the whole thickness of the line embraced by the ligature, by which this is also detached, and by which a portion of the vessel forms a real eschar. In an artery, if the ligature be not too large and is properly applied, a moderate degree of inflammation affects the vessel above and below it. A slight effusion of coagulable lymph takes place on its internal membrane, and adhesion by the first intention is the result; a coagulum of a conical figure, albuminous at its base and sanguineous at its summit, is found, near the adherence of the sides of the vessel, to oppose the effort of the blood; and, by eliminatory inflammation, the artery is gradually destroyed in the point correspondent to the ligature, which at length is detached. This happens from the tenth to the fifteenth day, if the vessel alone has been comprised in the fold of the thread, whatever may be its nature; but this is longer in taking place, if the thread does not act immediately on the vessel, and if it comprehends fibrous parts. The coagulum is not adherent to the internal membrane in the first few days; but, by an inflammation that takes place in the tissues of the vessel, it terminates by forming an adhesion.

"If any cause opposes itself to the union of the vascular parietes in the points adjacent to the ligature, and when the coagulum is not yet adherent, hæmorrhage may occur; and, if a ligature of reserve be employed, we easily divide the whole of the parietes of the artery in a point where the coagulum does not occupy the whole calibre of the vessel, and where it consequently cannot adhere to the internal membrane. This section of the arterial parietes by a ligature of reserve is effected more promptly in proportion as the inflammation has made the tissues of the artery lose their resistance, and permit themselves to be divided like parts affected with the sebaceous degenerescence. These observations on the mode of resistance of inflamed tissues, and on the true causes of consecutive hæmorrhages, appertain to M. Dupuytren. I have often had occasion to verify their justness and importance.

"If an hæmorrhage happens a short time after a ligature has been placed on an artery, it is not immediately above the first that a second should be applied, but on a distant point, where the arterial parietes are not inflamed. Has a large ligature been applied on an artery? the section of the vascular parietes has not been effected, and union by the first intention is not effected; it is the suppurative and ulcerative inflammation that then ensues; and, when the whole thickness of the vessel has been destroyed by this process, hæmorrhage is the consequence.

"It is not exactly thus with respect to the veins. Adhesive inflammation being excited with more difficulty in these vessels, and their internal membrane not offering the same facility of division as that of arteries, adhesion by the first intention does not take place, or only with great difficulty. The facility with which the tissues of veins are distended with blood, is another unfavourable circumstance to the primitive union. A more powerful and a more durable irritation is necessary to induce inflammation of veins. Albuminous matter alone is secreted if the inflammation be moderate, or else to this secretion is joined the formation of pus if the inflammation be intense. When the inflammation extends on one of the surfaces of the vessel, either from the force of the irritation that has acted on it, or because the pus has circulated in the part of the vein which is between the ligature and the heart; and it is, without doubt, to this propagation of the inflammation, that the development of general symptoms, the severity of the disease, and often the death of the subject, is to be attributed.

"The part of the vein situate near the ligature inflames; the membranes of the vessel acquire an inordinate thickness; the canal of the vein is filled up, and to a great extent, with a concrete albuminous matter, which soon adheres to the vascular parietes, and renders the vein impervious to the blood. The ulcerative and eliminatory inflammation then divide the whole thickness of the vessel in the circular line embraced by the ligature.

"*The treatment.*—It should be directed by the same principles as that of other inflammations. We should first endeavour to ascertain its cause; and, if it has happened after venesection, determine whether or not the instrument has been broken, and the point of it left in the parieties of the vessel.

"When the inflammation is solely local, we have been advised to put a stop to it suddenly, in its commencement, by cold lotions or fomentations,—as ice and saturnine applications. When the disease is more advanced, it should be combatted by the application of leeches on the course of the affected vessel; relaxing fomentations; emollient cataplasms; oily and mucilaginous unguents; tepid baths; camphorated, opiate, and narcotic, topics.

"J. Hunter, Reil, and Mr. Abernethy, think that we should, and that we may, incite adhesive inflammation, above and below the part affected, to oppose the extension of the disease, and prevent its extending further and further up the venous trunks, and to the heart itself. This adhesion would also prevent the pus, circulating with the blood, from irritating the internal membrane of the vessel over which it might flow. From what I previously said, it will appear that we cannot depend on the effects of compression in exciting this adhesion, because it is very difficult to obtain adhesive inflammation in the veins.

"A more certain mean would be to cut the affected vein through transversely, at a certain distance from the seat of the disease, to break the continuity of the tissues, and thus oppose the propagation of the inflammation. We may conceive that this operation is practicable only in cases where the affected vein is not very large in calibre, readily accessible to instruments, and when we need not fear the wounding of an artery or an important nerve.

"Prudence requires that, in the operation of phlebotomy, we always endeavour to obtain union by the first intention of all the different parts interested; and, if new emissions of blood are desired a short time after the first, that we do not destroy the process of primitive cicatrization to obtain blood by the same opening, but that we make another in a second part, and, if it be possible, in a different vein.

"If the inflammation has terminated by suppuration, we should, without delay, open the abscess to obtain a free course for the evacuation of the purulent matter.

"Lastly, if general symptoms appear, and they are intense, we should combat them by all the means directed for the treatment of angeio-tenique (inflammatory) fever and the phlegmasiæ."—*Journal comp. du Dict. des Sciences Medicales*, tome iii.

---

### *An Account of the Structure of the Brain and its Appendages;** by G. Lauth, Professor to the Faculty of Medicine at Strasbourg.

[From the *Journal comp. du Dict. des Sciences Medicales*, tome iii.]

The brain, or encephalon, is the organ contained in the cavity of the cranium, termed by the ancients the superior venter. In order

* The numerous discoveries which have been made during the last twenty years, respecting the brain, including those of Gall, has rendered necessary a new description of that organ, which should comprise the discoveries of the physiologist we have named, without being subservient to his doctrines. This task has been undertaken by Prof. Lauth, and we are convinced that our readers will contemplate with much interest the results of his labours.—Edit.

to describe it completely, we shall commence, by way of prolegomena, with an examination of its envelopments, a description of its different substances, and the denomination of its vessels. We shall also indicate the modes of dissecting it, and shew the divisions which we believe best adapted to favour the embracing in one view the numerous parts of which it is composed.

§ 1. *Prolegomena. The envelopments of the brain.*—The cranium is covered externally by the hairy scalp and the muscle termed the epicranium, which furnish to this osseous vase a thin envelopment, above, in front, on its sides, and on its posterior surface. The cranium and the brain itself are, in consequence, much exposed to the intemperature of the air, to the consequences of compression, to wounds, &c. The basis of the cranium is better guarded, being turned towards the neck; it is united to it by muscles and strong membranous ligaments, and there is also much adipose tissue interspersed between these parts. The cerebral envelopments within the cranium, are the *dura mater*, a strong, fibrous, white, and vascular, membrane, which serves at the same time for an internal pericranium, and gives out various prolongations; as, the falx, situated between the lateral parts of the brain; and the tentorium, placed between the cerebrum and cerebellum. The second membrane is the *arachnoidea*, a transparent lamina, thin and without blood-vessels, in contact with the pia mater over the whole of the surface of the brain, except at its basis, where it is a little separate from it, as well as over the spinal marrow. The *pia mater*, or the third cerebral envelopment, is a thin vascular membrane, which immediately embraces the cerebral substance, and which penetrates into its interior, either in dipping into its furrows, or in introducing itself by openings: it receives, in the last case, the name of the *plexus choroides*.

*The substances of the brain.*—In the embryo there is only a single cerebral substance, which, with the perfectionment of organization, is divided into two matters, distinguished by their colour and their structure. The first is *grey*, and termed the *cervical*, because it occupies the greatest part of the surface of the brain, and enters into its furrows, where it covers the lateral surfaces of its convolutions. The second is *white*, and bears the name of the *medullary* part, because it is often placed interiorly; although several organs of the brain are white on the surface and grey within. These two substances are sometimes distributed in layers, so that the external grey part is not continuous with the internal white matter.

These two substances present various shades of colour in different parts of the brain. Malacarne* long since made this remark. Not much attention has been paid to the tints of the white substance; but those which the grey presents have induced Sœmmerring† and Gennari‡ to admit a third, *yellow* substance; and a fourth, *black*. We, indeed, always find a blackish crescent in the crura of the brain; but the

* *Encefalotomia*, P. ii. No. 15.

† *De Basi Encephali*, page 63.

‡ *De peculiari Structura Cerebri*, p. 72.

yellow substance, which Sœmmering says is evident, particularly in that portion of the posterior lobe of the brain which rests on the tentorium of the cerebellum, and which Rolando* describes in the cerebellum, does not constantly exist, according to our own observations. There are some bodies where the deep-grey colour passes suddenly to white; we then do not perceive the yellow: in dead bodies, on the contrary, where the grey gradually becomes pale, it appears yellow before the commencement of the white colour; as Vicq-d'Azyr† also remarked. Gall‡ believes the grey substance to be diversely coloured and modified, according to the different destination of the nervous filaments which are thence derived.

The grey substance of the brain is the softest solid in the human body. We perceive in it fibres irregularly disposed, which are for the greater part blood-vessels; because it takes the colour of the matter of injections, when we have succeeded in well filling the vessels with the latter. This circumstance led Ruysch§ to imagine, that it was entirely vascular, and not formed of follicular glands, as Malpighi|| had believed. Albinus¶ modified the doctrine of Ruysch, saying that there existed a small quantity of grey substance incapable of being injected, interposed between the vessels, and forming the basis and the proper character of this part of the encephalic mass. The beautiful researches of Prochaska** shew also that, in the grey substance of the brain, as well as in all the other solid parts of the body, there is a portion which can never be injected. Anatomists have not hitherto been able to separate the grey pulp from the fibres that sustain it, probably in consequence of the softness of the latter. The grey substance, always uniform, but more vascular than the white, should be considered as the preparative organ of the latter.

The white substance does not change its tint by the nicest injections, because it does not contain a sufficient proportion of blood-vessels. It presents in almost every part a fibrous aspect, which has led to an opinion that a white fibre is the true element of its structure. This opinion corresponds with that of the ancients on the structure of the nerves, which they termed the third vascular system. They arrived by degrees to the hypothesis, that the red blood-vessels terminate, in the grey substance, in grey vessels, which themselves became white canals, composing the white substance and the nerves. This doctrine was particularly developed by Boerhaave.†† Modern anatomists no longer admit, it is true, a tubular structure in the white substance; but its fibrous formation appeared indubitable to Haller,‡‡

* *Saggio sulla Vera Struttura del Cervello,* p. 24.

† *Traité d'Anatomie et de Physiol.* Pt. ix. No. 10.

‡ *Anat. et Physiol. du Cerveau,* i. 234.

§ *Thesaurus Anat.* vi. 10, 38; ix. b.

|| *De Cortice Cerebri,* p. 1; *De Viscerum Structura,* p. 78.

¶ *Annotat. Academ.* i. 51.

** *Disquisitio Organismi Corporis Humani,* p. 103.

†† *Institut. Rei Medic.* No. 270, 274.

‡‡ *Elem. Physiol.* iv. 31.

MALACARNE,* SŒMMERING, MONRO,† VICQ-D'AZYR,‡ GALL,§ ROLANDO,|| REIL,¶ CARUS,** MECKEL,†† and WENZEL.‡‡

Notwithstanding these respectable authorities, it will be evident, from reflection on the ensuing details, that the fibrous aspect depends on the presence of a membrane, and that the white substance itself is pultaceous, semi-fluid, and not solid. We have seen it thus in trepanning a man who had received a gun-shot wound in the head, whose brain escaped through the fractured skull and torn membranes. This substance constantly presents the same appearance after death. When a nerve is cut across, it is driven out by the contraction of the tunics; it escapes from the white portion of the brain and from the spinal marrow, when the surface is slightly wounded. It receives its distinct figure from the membrane that sustains it, on which it is spread, like colour on the canvass forming the ground of a picture. Any person may be convinced of this, by an examination of the encephalon, the spinal marrow, the nerves, the retina, and the terminations of the auditory nerve. This fine membrane is indeed so well known, that we are surprised that authors have not recognized in it the general utility which we have indicated. Malacarne§§ speaks of an epithelium which envelops the white substance, and which is a continuation of the pia mater. Vicq-d'Azyr|||| found the tænia semicircularis covered by a firm, transparent, lamina. There is, according to Gall,¶¶ a neurilema situate between the two medullary strata which form each circumvolution of the brain. Reil*** speaks of a fine membrane situate on the surface of the medullary organs. KEUFFEL††† distinguishes in the spinal marrow, the white pultaceous substance from another, which is fibrous and more compact. Wenzel‡‡‡ says, that the ventricles are lined by a membrane. The white substance is then composed of two elements: one, pultaceous, which is the essential part of it; the other, membranous, which gives solidity to the former, and causes the fibrous appearance which this organ presents.

We cannot quit this subject without speaking of the opinion which is in opposition to what we have advanced, and without giving an exposition of the researches of modern anatomists on the intimate structure of the cerebral substances. Gall calls ganglions "the swellings composed of gelatinous substance, which is greyish in animals of the

---

* *Esposizione della Vera Strut. del Cervelleto*, No. 95.

† *Observations on the Nervous System*, page 25.

‡ *Traité d'Anat. et de Phys.* Pt. iv. x. &c.

§ *Anat. et Physiol. du Cerveau*, i. 237.

|| *Saggio della Vera Struttura del Cervello*, page 11.

¶ *Archiv. fur die Physiol.* ix. 148, 173.

** *Darstellung des Gehirns*, 39.

†† *Mensliche Anatomie*, i. 267, 278.

‡‡ *De Penitiore Structura Cerebri.*

§§ *Encefalotomia*, P. ii. No. 33, 35, 45, &c.

|||| *Traité d'Anat. et de Phys.* pl. v. vi. viii. ix.

¶¶ *Anat. et Phys. du Cerveau*, i. 298.

*** *Archiv. fur die Physiologie*, xi. 91, 101.

††† *Dissert. de Medulla Spinali*, § 15.

‡‡‡ *De Penit. Structura Cerebri*, p. 69, 85.

inferior class. The more considerable these masses are, (he says,) the more numerous are the nerves which spring from them. Now, these same conditions being present in animals of the highest class, we should regard these ganglions as the origin of the nerves. No nerve comes from another nerve; but each takes its origin in its proper mass of gelatinous substance, which is the matrix and nutritive matter of the nerve. Wherever the grey substance exists, we should suppose that there is the commencement of nerves; and he who commences his descriptions by nerves already formed, proceeds in a way that is contrary to the laws of nature. What is more conformable to reason than to commence investigations at the same point where nature began her work? It was not ascertained that the nervous fibres owed their origin and support to the grey substance. The fibrous structure of the white substance, (says this author,) should be recognized as a thing demonstrated: besides, the idea of its being a pulpy matter contradicts all that is known of anatomical and physiological phenomena." Gall nevertheless acknowledges, " that the brain is composed of fibres of such a degree of delicacy, that it may be only a uniform mass." The cortical substance tends, according to Reil,* to the globulous structure, and the medullary part to a radiated structure. Wenzel † believes almost all the substances of the human body to be formed of round corpuscules, which are formed of a common cellular substance, and of another proper to each organ, and enclosed in the cells. He considers, with respect to the brain, the grey substance as the seat of sensation and of the particular function of each organ, and the white appears to him to serve as a conductor. The matrix of the nervous parts is, according to Carus,‡ composed of globules, which are the type of animal organization, and are disposed in lines and in membranes. The cortical and medullary substances only differ in consequence of the globules being disposed in right lines in the latter, whilst in the former they constitute a thick tissue.

Our intention is not to refute the opinions of others, and still less to enter into discussion with any person whatever; we shall, therefore, confine ourselves to the following observations. Persons versed in the use of the compound microscope, see the elements of the human body, and of bodies in general, in a globular form, as DELLA TORRE;§ in a tortuous form, as FONTANA;|| in a serpentine form, as MONRO;¶ and it is ascertained that all those appearances are merely optical illusions: it is then superfluous to dwell on the consideration of elementary forms. With respect to the relation between the grey and white substances, we had previously recognized the extreme importance of the grey; and we know that BÖERHAAVE** said, an age since, that the white had its origin in the grey substance.

---

* *Archiv. fur die Physiologie*, ix. 487.

† *De Penit. Struct. Cereb.* 37, 69.

‡ *Darstellung des Gehirns*, 54, 59, 66.

§ *Nuovi Osservasioni Intorno sulla Storia Naturale*, page 144.

|| *Venin de la Vipère*, ii. 209, &c.

¶ *Observations on the Structure of the Nervous System*, tab. xxviii.

** *Institut. Rei Med.* § 237.

*The vessels of the brain.*—The blood-vessels, arterial and venous, are disposed in a particular manner in the organs under consideration. The encephalic arteries* are derived from the internal carotid and vertebral arteries: the latter unite into a single trunk, the basilary artery, the branches of which form, with those of the carotids, the arterial circle of Willis, so that there exists a communication between all the encephalic arteries. The spinal marrow receives its branches from the vertebral, dorsal, lumbar, and sacral, arteries. These vessels carry with them the pia mater into the cerebral substances: they traverse, in the hemispheres of the cerebrum, and in the lobes of the cerebellum, the grey substance to penetrate into the white substance; but, in the corpus callosum, and in the crura of the cerebrum, the white substance is the first which receives them. The final arterial ramifications are so delicate, that they have not yet been filled with the matter of injections, which in the brain does not return by the veins. The latter† are formed by very thin tunics; they end in the sinuses placed between the laminæ of the dura mater, of which those that receive the cerebral veins terminate in the internal jugular veins; the sinuses of the spinal marrow discharge themselves into the vertebral, dorsal, lumbar, and sacral, veins.

Lymphatic vessels have not yet been perceived in the brain. Monro‡ considered that it must possess them. Cruickshanks§ detected a lymphatic gland in the carotidian canal. He thence concluded that lymphatic vessels existed in the brain. Mascagni‖ described and figured lymphatics in the membranes of that organ, but he could not perceive them in the cerebral substances.

*Dissection of the Brain.*—There are several modes of dissecting the brain: one consists in removing the substances by horizontal and oblique slices; this method bears the name of Vesalius when it is practised from the upper to the inferior part, and that of Varolius when we proceed from below upwards. The other mode of operating, that of scraping the brain with the edge of the scalpel, is necessary for the perfect development of the different parts of this organ. Malacarne and Gall recommended it. Both of them have practised it on the fresh brain.

There are some circumstances in which it will be necessary to harden the brain before it is examined. This may be effected either by boiling it in oil, or by macerating it for several days in alcohol, or a solution of corrosive sublimate or of alum, and afterwards plunging it for several hours in pure water, to diminish its too-great rigidity. The brain thus prepared is torn with the fingers and the handle of the scalpel, in following the direction of the apparent fibres. Reil points out the mode of proceeding on this occasion.

---

* Haller, *Icones Anat. Fasc.* vii.; Vicq-d'Azyr, *Traite d'Anat. et de Physiol.* pl. xix.

† Vieussens, *Neurologia*, liv. ii. chap. 2; G. Lauth, *Spicilegium de Vena Cava Superiori*, p. 74.

‡ *Observations on the Structure of the Nervous System*, p. 17.

§ *The Anatomy of the Absorbing Vessels*, page 185.

‖ *Vasorum Lymphat. Historia*, p. 65; tab. xxvii. f. 1, 2, 3.

We cannot always do without the assistance of optical instruments; but, in these cases, a good simple convex glass is preferable to the compound microscope, because of the illusions of the latter, against which we cannot take too many precautions.

*Division of the Brain.*—The brain is divided into a superior and most considerable portion, termed the *cerebrum;* and the *cerebellum,* which is the small portion placed inferiorly and posteriorly.

We shall commence the examination of the cerebrum by its surface, superior as well as inferior, on which we find numerous furrows and three openings which lead into the interior cavity of the organ. We shall give to the whole of these objects the name of the *peripherical arrangement;* and we shall treat particularly of the hemispheres, the circumvolutions, the fissure of Sylvius, the ventricles, and the plexus choroides. These parts form about the half of the cerebral mass.

When the hemispheres are removed, the *centrum ovale* and the *corpus callosum* become exposed to view. If we cut through the centrum ovale at a short distance from the corpus callosum, we perceive that its inferior surface is continuous with the *septum lucidum,* which rests on the fornix, itself supported by four pillars, two situated anteriorly and two posteriorly. The latter accompany the *cornua ammonis,* which are themselves the continuation of the two angles of the posterior extremity of the corpus callosum. Each of those cornua descends in the inferior horn of the lateral ventricle of the same side, in the course of an inclined plane from behind forward, which cuts the middle lobe of the cerebrum into two parts by its diagonal. Thus, the corpus callosum, the septum lucidum, the fornix and its pillars, the cornua ammonis, the inferior portion of the middle lobe, and the posterior lobe of the cerebrum, are especially connected together, and form what we term the *department of the corpus callosum.*

The portion of the cerebrum which remains after the separation of the department of the corpus callosum, appertains to the *department of the crura cerebri,* where we find the corpora striati; the tænia semicircularis; the thalami optici; the third ventricle; the two commissures, anterior and posterior; the crura cerebi; the anterior lobes, and the superior portion of the middle lobes.

The pituitary gland and infundibulum are parts which should be separately considered. They form an *appendage* to the cerebrum.

The other portion of the encephalic mass is the *cerebellum,* situate under the tentorium, in the occipital fossa. We here observe the lobules, the processus vermiformis, the corpora striata, the crura cerebelli, the arbor vitæ, and the pedunculi.

After the cerebrum and the cerebellum, we shall separately consider the *pons Varolii,* because it does not appertain particularly to either of those two organs, but the substances of both of them enter into its composition. We shall call it the *pons inferior,* to distinguish it from the *pons superior,* which is commonly known under the name of the *tubercula quadrigemina,* which is more especially a pons than that of Varolius, and which, like the latter, is composed of the substances of the cerebrum and cerebellum. The pons Varolii is situate at the basis of the cerebrum, near the centre of the basis of the cranium. The superior pons occupies the centre between the cerebrum and cerebellum.

The *medulla oblongata*, which rests on the basillary fossa, forms a continuation of the two pontes, as being connected to the posterior part both of the pons Varolii and the tubercula quadrigemina. It is continuous with the *medulla spinalis*, which is enclosed in the vertebral canal.

The history of the encephalon and the medulla spinalis comprises also the description of the origin and of the structure of the *nerves*, which leave the cranium and the vertebral canal to be distributed throughout the body.

These organs exist for the most part in pairs, and are placed on each side of the axis of the body. The impairal nerves occupy the axis itself, and are divided into two equal lateral portions. All these parts, pairal as well as impairal, have consequently a symmetrical structure.

The subject under consideration will therefore be distributed in the following manner:

1, The cerebrum, *(peripherical arrangement; department of the corpus callosum; department of the crura cerebri; appendix of the cerebrum;)* 2, the cerebellum; 3, the pontes; 4, the medulla oblongata; 5, the spinal marrow; 6, the nerves.

*(To be continued.)*

---

### *A Memoir on Ileus, and on a particular Method of Treating it.* By J. D. Brandis, Member of the Royal Society of Medicine of Copenhagen.

[From the *Nova Acta Regiæ Societatis Medicæ Hauniensis*, 1818; as abridged in the *Nouveau Journal de Medecine.*]

"In the year 1794, being at Brunswick, where I was practising medicine, I was called to a patient, who was attacked eleven days previously with ileus, and in whom the symptoms were most alarming,—such as delirium, coldness of the extremities, hiccup, facies hippocratica; leading me to fear the near approach of death, and leaving less reason for hope, from many remedies having been used by physicians of eminent abilities. I recollected the method of treatment that Frederick Hoffmann had once seen employed by Naboth, and to the use of which he had only consented by a sort of condescension. The result was more fortunate than Hoffmann had expected. On cold water being administered several times a-day, in doses of two glasses, the trunk and feet being previously well covered, a copious sweat broke out, to which succeeded a tranquil sleep; and the pain in the bowels, as well as the vomiting, had ceased. Naboth moreover stated, that he employed with success, in three cases, compresses moistened with cold water over the abdomen. Many other physicians, as De Haen, Chavasse, Stoll, and Van Swieten, had also obtained good effects from those means, in analogous cases. I consequently determined to have recourse to them in the one that was presented to me. I prescribed the use of an iced drink, and had the belly covered with cloths dipped in iced water. In a few hours the delirium ceased: twentyfour hours afterwards the extremities had recovered their natural heat, the hiccup was less frequent, the vomiting more rarely occurred, and gradually entirely ceased. Yet the constipation continued, notwithstanding the use of cold and warm enemas frequently repeated. The appetite was almost extinct, and the patient only took a little animal jelly mixed with ice. I prescribed opium in small doses, and the decoction of cinchona also mixed with ice. The patient continued about seven days in this state. During this time the omission of the cold fomentations to the abdomen constantly gave rise to returns of the vomiting; so the patient himself desired their re-application. At length, on the seventh day, a copious diarrhœa came on: the cold fomentations were then suppressed, having become useless; and in the space of four days, with the aid of cold and nutritive food, the re-establishment of health was complete.

"After such unexpected success, I placed my whole confidence in this measure, and my hope has never been deceived. I have applied cold fomentation on the abdomen of delicate women as well as of robust men, and I have found in it a sure and prompt remedy. I have used it in a man 68 years of age, on the eighth day from an attack of ileus, which left nothing to hope. This old man was cured, although there was in the inguinal ring a small and immobile herniary tumor, with gangrene of the portion of the epiploon, and the formation of an abscess between the abdominal muscles.

"In 1814 I had, for the tenth time, occasion to experience the good effects of this method, conjointly with our celebrated Callisen and Dr. Strom.

"A woman, 22 years of age, the mother of two children, was subject to spasms of the intestines, vomitings, and colics, at every menstrual period. She was attacked with ileus in the month of January, after exposure of the feet to cold, towards the epoch of menstruation. She not only vomited all she swallowed, but enemas, prepared with assafœtida, were also thus rejected: to this symptom were added coldness of the extremities, obstinate hiccup, and a frequent small pulse, that led us to presage a fatal termination. After the sixth day I prescribed, in the first instance, some glasses of iced water and the tinctura thebaiaca, combined with two parts of the essence of castoreum. Four hours afterwards there was no amendment; the medicines had been rejected, and the symptoms were still more severe. I prescribed cold fomentations, and increased the dose of the anodyne tincture to thirty drops.

"Six hours afterwards, the vomiting had only once occurred; the hiccup was less fatiguing, the extremities were warm. I directed neither glysters nor any other remedy proper to provoke evacuations, from the fear of exciting anti-peristaltic contractions of the large intestines. I continued the use of the same means for four days: on the sixth day of this treatment the bowels were spontaneously opened, and the patient became convalescent.

"It is of the greatest importance in the administration of this remedy, to insist on it with perseverance. In the first patient, the use of it was continued for nine days; in two others, during twenty-four hours. I have had recourse, with success, to the same remedy in many other affections, and particularly in very violent colics, and in dysentery without fever.

"The good effects I have obtained from the use of ice in ileus, would not lead me to advise it in all cases without discrimination, and to the exclusion of all other means. I have seen, in one case, the extraction of a carious tooth put a stop to all the symptoms of ileus. A woman, about 20 years of age, was taken, at the time of her menses, with a very violent tooth-ach: some person advised to place a piece of very cold iron between the gums and the cheek; the pain disappeared; but at the same time the belly became painful, the extremities cold; the patient was seized with continual vomiting, and the alvine evacuations were suspended. Being called on the third day, I prescribed the use of ice, but with little advantage. The manner in which the disease had first appeared, furnished a special indication: the tooth was drawn; all the symptoms were suddenly alleviated, and a few hours afterwards the bowels were opened without the aid of medicine.

