# Errata

**Published:** 1819-11

**Authors:** 


*** *We request our readers to correct an error in a calculation of the relative mortality from parturition and its express consequences, which appeared in the 256th page of this volume. That calculation was made from partial statistical accounts, and chiefly from Hospital records in different parts of Europe. On a more full and correct enquiry, we find it to be in the proportion of about one in one hundred. According to the Bills of Mortality, the proportion in* London *for the last year was one in* 109; *and this has been nearly the average proportion during the last twenty or thirty years.*

*Read, also, page 237, 6 lines from the bottom,* perceptible *for* forcible.

